# Platypnea-orthodeoxia syndrome in COVID-19: rare, not uncommon

**DOI:** 10.1186/s43168-022-00124-y

**Published:** 2022-05-09

**Authors:** Renu Kumawat, Mahesh Lal, Jagadeesan S., Prarit Varshney

**Affiliations:** 1 Department of Internal Medicine, Vardhman Mahavir Medical College & Safdarjung Hospital, New Delhi, 110029 India; 2 Guru Gobind and Indraprasta University, New Delhi, 110029 India

**Keywords:** COVID-19, Platypnea-orthodeoxia syndrome, Oximetry

## Abstract

*COVID*-*19* is a contagious disease caused by the severe acute respiratory syndrome coronavirus-2 (SARS-CoV-2). As humanity continues to fight this virus, it keeps presenting with new manifestations and complications. Platypnea-orthodeoxia syndrome (POS) is a rare clinical syndrome characterized by dyspnea and oxygen desaturation, in the supine to sitting position, which resolves with recumbency and can be diagnosed with a simple pulse oximeter. We report three cases of COVID-19 pneumonia, who developed POS during the recovery phase, in the absence of pulmonary hypertension.

## Background

Platypnea-orthodeoxia syndrome (POS) was first described in the 1940 [1], but the patho-physiological mechanisms are still not completely understood. It is characterized by orthostatic dyspnea and a fall in arterial oxygen saturation of > 5% or a PaO_2_> 4 mmHg [2]. The condition has been reported in cardiac and pulmonary conditions, with a right to left shunt.

The spectrum of severity of COVID-19 varies widely from mild respiratory disease to pneumonia with severe acute respiratory distress syndrome (ARDS), necessitating invasive mechanical ventilation. Here, we report three cases of POS in the recovery phases of COVID-19, who improved with oxygen and physiotherapy. It is imperative for the clinician to be aware of this condition, which can be easily diagnosed bedside.

## Case report 1

**Fig. 1:**
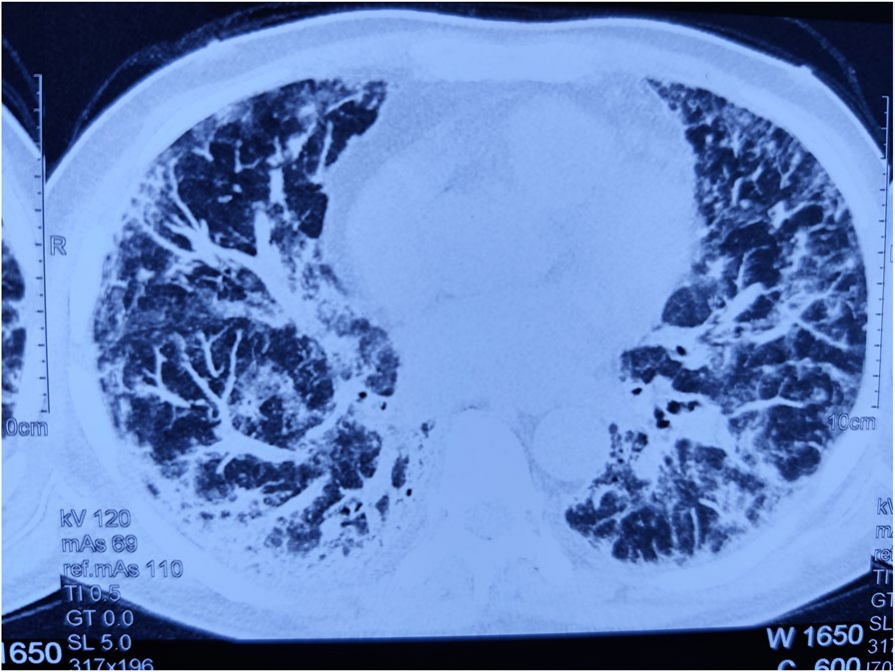
HRCT-Chest showing diffuse areas of ground glass opacities with interlobular septal thickening in bilateral lung fields

A 50-year-old male presented with high-grade fever and breathlessness for the preceding five days. On examination, he had an oxygen saturation of 84% on room air. Arterial blood gas analysis showed pH 7.43, pCO_2_ 23 mmHg, pO_2_ 48 mmHg. Chest X-ray showed bilateral radio-opacities with lower zone predominance. HRCT-Thorax revealed diffuse ground glass opacities with interlobular septal thickening in both lung fields with a CT severity score [3] of 24/25 (Fig. 1). Nasopharyngeal swab for SARS-COV-2 RT-PCR was positive on the next day. Inflammatory biomarkers were as follows: CRP 48 mg/L, Ferritin 386 ng/mL, LDH 630 U/L, and IL6-176 pg/mL.

Patient was diagnosed with severe COVID-19 pneumonia and was shifted to the ICU with high flow oxygen support at 70 L/min using a high flow nasal canula and was treated with parentral methyl prednisolone 1–2 mg/kg intravenous in two divided doses for 10 days and parentral enoxaparin 0.5 mg/kg per dose subcutaneously 12 hourly for 2 weeks. After 2 weeks, repeat COVID-19 RT-PCR turned negative and his inflammatory markers decreased to almost normal levels. As his oxygen saturation was about 90% on room air, he was shifted out of ICU and kept on low flow oxygen and bedside chest physiotherapy.

During this recovery phase, the patient repeatedly complained of uneasiness and shortness of breath, shortly whenever he tried to sit in his bed. The complaints and findings were consistent and reproducible. His oxygen saturation was checked in the lying and sitting positions, which were 94% and 86%, respectively, confirming a diagnosis of POS.

His echocardiography was normal with no evidence of pulmonary hypertension, systolic, and diastolic dysfunction. All chambers were normal with no evidence of any clot or vegetations or any other morphological abnormalities. However, cardiac catheterization could not be performed due to ongoing pandemic. He improved with low flow oxygen at 2–3 l/min and chest physiotherapy for 2 months and was discharged on oxygen saturation > 96% on room air to follow-up in the post-COVID clinic and doing well at present.

## Case report 2

**Fig. 2:**
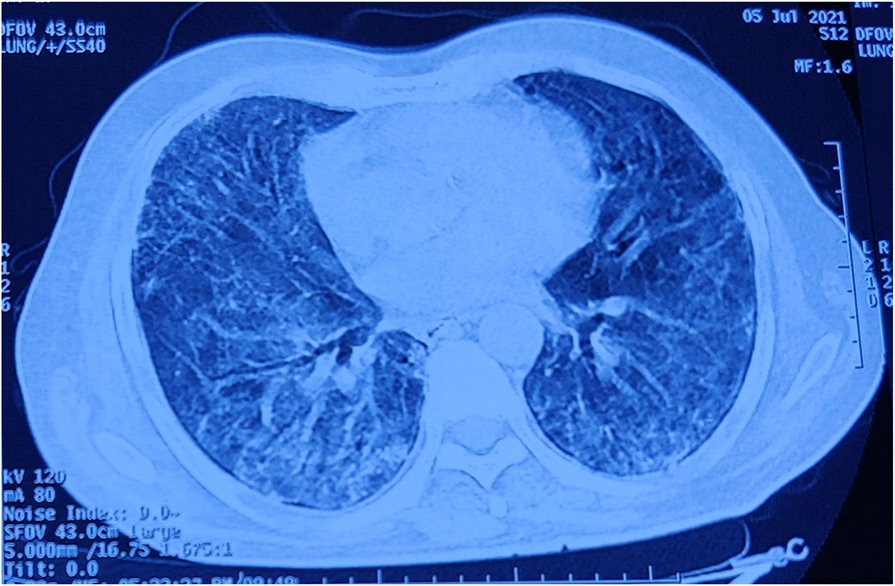
HRCT-Chest showing diffuse areas of ground glass opacities and non-specific interstitial pneumonia

A 52-year-old man presented with high grade fever and shortness of breath for 7 days. One month back, he was diagnosed as mild COVID-19 pneumonia, for which he was treated with parentral methyl prednisolone 1–2 mg/kg intravenous in two divided doses for 10 days and parentral enoxaparin 0.5 mg/kg per dose subcutaneously 12 hourly for 2 weeks. On admission, his O_2_ saturation was 74%. ABG analysis at room air revealed: pH 7.43, pCO_2_ 34 mmHg, pO_2_ 45 mmHg, Chest X-ray showed bilateral opacities with lower zone predominance and HRCT-Thorax revealed diffuse areas of ground glass opacities and non-specific interstitial pneumonia (Fig. 2). A nasopharyngeal swab for RT-PCR was negative. Inflammatory biomarkers had the following values: CRP 10 mg/L, Ferritin 302 ng/mL, and IL6 32 pg/mL.

He was initially kept on oxygen support by non-rebreather mask at 10 L/min, which gradually decreased to 2 L/min. After a few days, the patient started complaining of increased shortness of breathlessness on sitting. His oxygen saturation in the lying and sitting positions, were 95% and 87%, respectively, confirming a diagnosis of POS.

His echocardiography was normal. However, cardiac catheterization could not be performed due to ongoing pandemic. He improved with low flow oxygen at 3–4 l/min and chest physiotherapy for 40 days and was discharged on oxygen saturation > 96% on room air.

## Case report 3

**Fig. 3:**
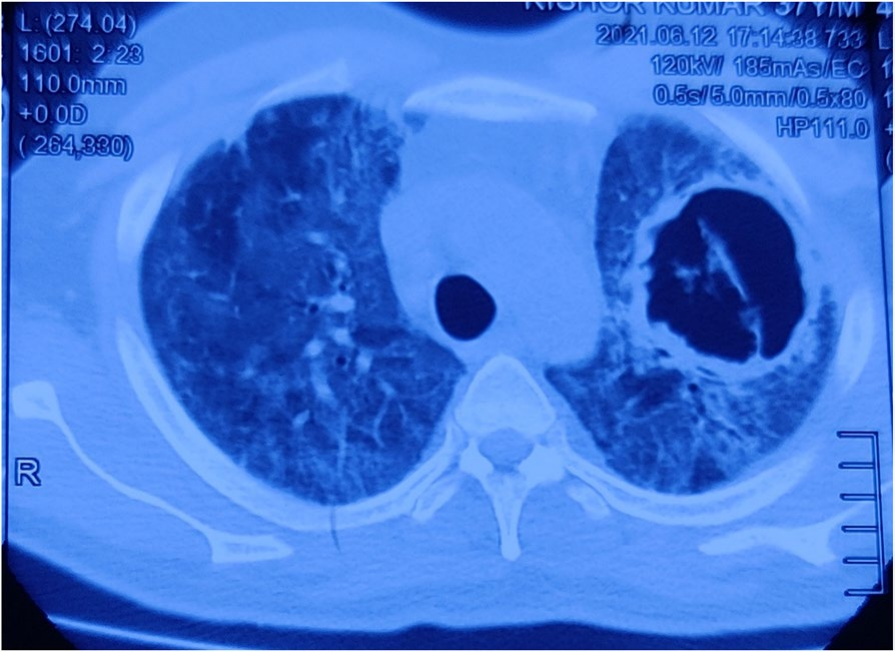
HRCT-Chest showing diffuse areas of ground glass opacities with interlobular septal thickening in bilateral lung fields and left upper zone cavitary lesion

A 37-year-old male, presented with complaints of fever, dry cough and shortness of breath for the preceding 10 days. His oxygen saturation was 85% on room air. Examination revealed bilateral diffuse fine end inspiratory crackles. HRCT-Thorax was suggestive of severe COVID-19 pneumonia with CT severity index of 25/25 (Fig. 3). The diagnosis of COVID-19 was confirmed by nasopharyngeal throat swab RT-PCR. He was treated with parentral methyl prednisolone 1–2 mg/kg intravenous in two divided doses for 10 days and parentral enoxaparin 0.5 mg/kg per dose subcutaneously 12 hourly for 2 weeks**.** Over the next 2 weeks, his fever subsided, the oxygen requirement decreased from 10 L/min to 4 L/min, and repeat RT-PCR was negative.

During further course, his fever relapsed and repeat investigations revealed: Hb-13.5 gm%, TLC 12,300 cells/mm^3^, platelets 3,54,000/mm^3^. ABG analysis at room air showed pH 7.42, pCO_2_ 25 mmHg, pO_2_ 52 mmHg. Repeat HRCT-Thorax revealed diffuse areas of ground glass opacities with interlobular septal thickening in both lung fields and a left upper zone cavitary lesion but nasopharyngeal swab for RT-PCR COVID-19 was negative. Inflammatory biomarkers had the following values: CRP 48 mg/L, Ferritin-390 ng/mL and IL6 93 pg/mL. As serum galactomannan test was positive and sputum for fungal culture showed growth of septate hyphae, he was started on parentral amphotericin B deoxycholate therapy.

During this recovery phase, the patient repeatedly complained of shortness of breath after he tried to sit up. His oxygen saturation in the lying and sitting positions, were 95% and 82% respectively, confirming POS.

His echocardiography was normal. However, cardiac catheterization could not be performed due to ongoing pandemic. He improved with low flow oxygen at 2–3 l/min and chest physiotherapy for 45 days and was discharged on oxygen saturation > 96% on room air.

## Discussion

POS is a rare clinical entity characterized by dyspnea (platypnea) and oxygen desaturation (orthodeoxia) in the supine to sitting position, which resolves quickly with recumbency. This drop in saturation is defined as a drop in PaO_2_ > 4 mmHg or SaO_2_ > 5% from supine to an upright position.

The primary etiologies of POS can be broadly classified as intra-cardiac or extra-cardiac. A patent foramen ovale (PFO) is the most common cause, followed by atrial septal defect (ASD) in the presence of pulmonary hypertension [2]. Extra-cardiac causes of POS include intra-pulmonary arterio-venous malformations and parenchymal lung diseases. Miscellaneous causes reported are pneumonia caused by pneumocystis jiroveci/cytomegalovirus pneumonia [4] and hepato-pulmonary syndrome [5].

While the commonest mechanism of POS in cardiac pathologies is an inter-atrial shunt, the patho-physiology of respiratory disorders differs. The proposed mechanism of POS in COVID-19 is gravitational dependent shifting of blood to the lower zones, leading to wasting of perfusion as posterior and lower parenchymal involvement is common in COVID-19 pneumonia, causing V/Q mismatch. Alternatively, it may be due to diminished perfusion in the lower zones, due to vasculopathy and coagulopathy [6]**,** or it may be the result of myocardial dysfunction [7, 8]. Factors that decrease cardiac output could contribute to further reduction of blood flow in the areas of non-dependent zone of the lungs, thereby worsening POS [9]. However, why this occurs particularly in recovery phase and also, the definite patho-physiology remains less understood. There is a possibility that it could be due to late recovery of the basal part of the lung or fibrotic changes in the basal parenchyma. However, definite pathophysiology is less understood at this point and this could be one aspect for future research studies.

POS in our patients was reversible with oxygen and physiotherapy, in concordance with few other reported cases from the country [6–8]. These cases have been reported to emphasize the importance of being vigilant in the recovery phase of COVID-19 patients, as underlying hypoxia may be missed, if only the standard supine method is employed to assess oxygen saturation.

## Conclusion

POS should be considered as a cause of respiratory impairment in patients of COVID-19 pneumonia after ruling out other causes. This case series has been reported to emphasize that POS may not be as uncommon as thought to be and high level of suspicion should be kept in COVID-19 patients, requiring prolonged oxygen support, especially those having positional exacerbation of dyspnoea.

## Data Availability

Yes.
